# Comprehensive Pain Management Using Opioids for Children and Adolescents: Still a Wild Goose to Chase?

**DOI:** 10.3390/children9030347

**Published:** 2022-03-03

**Authors:** Johanna M. C. Blom, Cristina Benatti

**Affiliations:** 1 Department of Biomedical, Metabolic and Neural Sciences, University of Modena and Reggio Emilia, 41125 Modena, Italy; 2 Center for Neuroscience and Neurotechnology, University of Modena and Reggio Emilia, 41125 Modena, Italy; cristina.benatti@unimore.it; 3 Department of Life Sciences, University of Modena and Reggio Emilia, 41125 Modena, Italy

Children and adolescents treated for acute and chronic pain represent particular vulnerable patients with distinct and unmet medical and psychosocial needs that continue even beyond the complexity of treating the diseases they suffer.

Severe acute and chronic pain during development interfere with cognitive functioning and educational achievement, induce changes in mood and behavior, influence peer relationships, and enhance the susceptibility for the onset of psychopathology [1]. Moreover, specific treatments are hampered by inadequate knowledge of safety and tolerability in this patient population and are often accompanied by numerous side effects such as sleep disturbance, fatigue, changes in body weight, nausea, vomiting, and treatment-induced depression and psychosis.

However, many exceptional efforts have shifted pain management from the exclusive realm of pharmacological treatment to a multidisciplinary approach based on the integration of non-pharmacological methods and psychotherapy. This resulted in ready-to-use toolboxes and harmonization of guidelines. Such an integrative multidisciplinary approach is even more important considering the possibility of non-pharmacological methods to reduce the overall use or dosage of opioids [2]. In their review, Wren et al. give an excellent account of the need for integrating non-pharmacological methods, their mechanisms of action, and their role in reducing the prescribing of opioids to pediatric patients. Even though large-scale efforts and integrated approaches have become the standard of care rather than the exception, many of the typical concerns remain largely unattended. This is partly due to not having been able to remove some persistent biases and barriers related to treatment and assessment, monitoring, and regulation.

Pain is a complex and highly subjective and individualized experience. For children and adolescents, both the assessment and management of pain can be a real challenge. Therefore, it is vital to capture the emotional, behavioral, and psychological changes as well as the biological alterations in young patients with pain as timely as possible. Most importantly, guidelines and reviews based on best practice emphasize the fact that pain experienced during development does not lead to negative chronic outcomes, especially if we know which individuals are at enhanced risk for opioid treatment related adverse effects. Adequate recognition and use of indicators of pediatric pain as well as verifying and monitoring the efficacy of treatment must be accompanied by various levels of analysis including biological, genetic, and behavioral data [3]. Consequently, if we want to detect the distinct clinical pathways of high-risk individuals as early as possible, a paradigm shift is essential. Only then will we be able to offer a genuinely integrated approach to pain management with non-pharmacological intervention being part of treatments mitigating the problems related to the use of opioids.

The quantification of pain is an issue in all populations, especially so in pediatric patients with pain that represent an enormously heterogenous group. While self-reporting remains the gold standard for pain perception and localization [4], assessment in children and adolescents suffering from acute and chronic pain must incorporate all levels of change from those observed at the biological level to changes related to emotion and behavior. Only in this way will we expand our understanding of the factors influencing their current and future wellbeing and to predict the immediate and lasting consequences. Innovative digital approaches to the monitoring and the care of patients offer an excellent opportunity to create predictive models of individual vulnerability based on the integration and interdependencies of diverse sources of information. Their analysis, then, allows the prediction of potential outcomes and disease trajectories, identifying those patients that are progressing from basic or moderate vulnerability for disease or treatment related negative events to an elevated risk [5]. Wren and coworkers suggest that non-pharmacological therapies provide the patients with a sense of “control” over their condition [2]: children and adolescents who can self-manage their pain demonstrate fewer depressive symptoms, fear of pain, functional disability, and negative coping strategies.

Youth express the need to be directly involved in their health and wellbeing and to have an active role that truly impacts the development of pain treatments [6]. When asked, adolescents indicate two areas of concern: (1) behavioral, emotional, physical, and mood problems related to pain; and (2) the need to be more connected, involved, and informed. In order to intervene in a timely and preventive manner, all problems in these realms of functioning have to be addressed.

Digital tools are noninvasive, ecological, and allow continuous access which provides timely appreciation of treatment induced changes, as well as emotional, behavioral, and cognitive alterations. A digital toolbox has numerous benefits compared to traditional assessments and represents an actual opportunity to modify, replace, or accompany the existing more categorical and traditional methods [5]. Gathering evidence from different domains, ranging from biomarkers to subtle neurocognitive evidence, will allow a shift to a truly comprehensive multimodal dimensional methodology [7]. Ultimately, the multidimensional continuous recording of data and especially their dynamic relationships together with biological predisposition (genetic expression of hepatic metabolic enzymes involved in opioid effectiveness or toxicity) may become an integral part of care plan and serve as an indicator of high-risk.

What we need now is to seize the moment and develop data-driven insights to deliver the right care to the right patient at the right time through regulatory innovation and scientific rigor. The improvement of pediatric drug development is especially important in pain management [8]: national and international guidelines are available and regulatory agencies are issuing pediatric regulation, such as the Best Pharmaceuticals for Children Act and the Pediatric Research Equity Act in the US and the Pediatric Regulation in the European Union. Since its introduction, European Medicines Agency (EMA) authorized more than 260 new medicines (both new marketing authorizations and indications) for use in children from 2007 until 2015 [9].

Real World Evidence (RWE) must be included in the ongoing discussions and initiatives in Pediatrics. In RWE, data derived from numerous sources measure both objective and subjective outcomes from the child’s home environment, school, and clinical setting. We need a holistic solution which combines data from different systems (including data from electronic medical records, lab data, physician transcriptions and prescriptions of opioids, non-pharmacological interventions, patient monitoring devices, and self-reports). Regulators should step up to the challenge and help to create the tools and ethical and legal infrastructure necessary to analyze and process this enormous quantity of data.

Notwithstanding the promise to deliver a revolution in the management of acute and chronic pain of children and adolescents and to avoid an everlasting wild goose chase, the incredible potential of the digital tools coming of age is accompanied by important questions regarding its real-world implementation.

The analysis of large multi-domain data needs a multidisciplinary collaborative effort which is largely lacking. A profound change in data gathering and integration is necessary: information coming from multiple sources including non-pharmacological methods and digital tools must be managed in a thoughtful way. Quality measures must be incorporated, and the sensitivity, specificity, accuracy, and precision of methods, measurements, and device parameters need to be tested [5].

A trusted ethical, legal, and regulatory ecosystem should be created to allow the involvement of young patients as data generators. Their sensitive, health-related personal data have to be shared in an ethical and privacy-compliant environment, built through a collective effort encompassing innovative, legal, organizational, and technical solutions.

Hence, embracing a holistic multidisciplinary and multidomain approach should be sustained by suitable regulatory support to totally protect the child and adolescent patient and to surmount present and future challenges related to confidentiality and accountability. Having the technical capacities and the knowledge to really profit from integrating new tools and methods, education, and training is needed both for the young patient and the clinician. Researchers and clinicians must avoid becoming the rate limiting step and move from “knowing by doing” to “doing by knowing” to make pain assessment and treatment a fully knowledge-based therapeutic area of intervention especially, but not only, in younger populations [10].

## References

[1] Lau J.Y.F., Heathcote L.C., Beale S., Gray S., Jacobs K., Wilkinson N., Crombez G. (2018). Cognitive Biases in Children and Adolescents with Chronic Pain: A Review of Findings and a Call for Developmental Research. J. Pain.

[2] Wren A.A., Ross A.C., D’Souza G., Almgren C., Feinstein A., Marshall A., Golianu B. (2019). Multidisciplinary Pain Management for Pediatric Patients with Acute and Chronic Pain: A Foundational Treatment Approach When Prescribing Opioids. Children.

[3] Eerdekens M., Beuter C., Lefeber C., van den Anker J. (2019). The Challenge of Developing Pain Medications for Children: Therapeutic Needs and Future Perspectives. J. Pain Res..

[4] Vega E., Beaulieu Y., Gauvin R., Ferland C., Stabile S., Pitt R., Gonzalez Cardenas V.H., Ingelmo P.M. (2018). Chronic Non-Cancer Pain in Children: We Have a Problem, but Also Solutions. Minerva Anestesiol..

[5] Blom J.M.C., Colliva C., Benatti C., Tascedda F., Pani L. (2021). Digital Phenotyping and Dynamic Monitoring of Adolescents Treated for Cancer to Guide Intervention: Embracing a New Era. Front. Oncol..

[6] Hunter J.F., Kain Z.N., Fortier M.A. (2019). Pain Relief in the Palm of Your Hand: Harnessing Mobile Health to Manage Pediatric Pain. Paediatr. Anaesth..

[7] Fisher E., Law E., Dudeney J., Eccleston C., Palermo T.M. (2019). Psychological Therapies (Remotely Delivered) for the Management of Chronic and Recurrent Pain in Children and Adolescents. Cochrane Database Syst. Rev..

[8] Walco G.A., Kopecky E.A., Weisman S.J., Stinson J., Stevens B., Desjardins P.J., Berde C.B., Krane E.J., Anand K.J.S., Yaster M. (2018). Clinical Trial Designs and Models for Analgesic Medications for Acute Pain in Neonates, Infants, Toddlers, Children, and Adolescents: ACTTION Recommendations. Pain.

[9] Tomasi: Enabling Development of Paediatric Medicines—Google Scholar. https://scholar.google.com/scholar_lookup?publication_year=2017&title=State+of+Paediatric+Medicines+in+the+EU-+10+Years+of+the+EU+Paediatric+Regulation.

[10] Eccleston C., Fisher E., Howard R.F., Slater R., Forgeron P., Palermo T.M., Birnie K.A., Anderson B.J., Chambers C.T., Crombez G. (2021). Delivering Transformative Action in Paediatric Pain: A Lancet Child & Adolescent Health Commission. Lancet Child Adolesc. Health.

